# Symbolic number comparison and number priming do not rely on the same mechanism

**DOI:** 10.3758/s13423-022-02108-x

**Published:** 2022-05-03

**Authors:** Attila Krajcsi, Tamás Szűcs

**Affiliations:** Department of Cognitive Psychology, Institute of Psychology, ELTE Eötvös Loránd University, Budapest, Hungary

**Keywords:** Approximate number system, Discrete semantic system, Comparison distance effect, Priming distance effect

## Abstract

In elementary symbolic number processing, the comparison distance effect (in a comparison task, the task is more difficult with smaller numerical distance between the values) and the priming distance effect (in a number processing task, actual number is easier to process with a numerically close previous number) are two essential phenomena. While a dominant model, the approximate number system model, assumes that the two effects rely on the same mechanism, some other models, such as the discrete semantic system model, assume that the two effects are rooted in different generators. In a correlational study, here we investigate the relation of the two effects. Critically, the reliability of the effects is considered; therefore, a possible null result cannot be attributed to the attenuation of low reliability. The results showed no strong correlation between the two effects, even though appropriate reliabilities were provided. These results confirm the models of elementary number processing that assume distinct mechanisms behind number comparison and number priming.

## Introduction

In the field of numerical cognition, the approximate number system (ANS) is believed to be a fundamental representation to understand and solve mathematical problems. For example, it was proposed that higher sensitivity of the ANS is related to better math performance in school (Halberda et al., 2008; Schneider et al., 2017), the representation may play a role in the initial acquisition of the symbolic numbers (Mussolin et al., 2012; Piazza, 2010; Rousselle et al., 2004; Wagner & Johnson, 2011), or the impairment of this system may lead to developmental dyscalculia, a math-specific learning deficit (Molko et al., 2003; Piazza et al., 2010; Price et al., 2007).

The ANS is a number representation that obeys Weber’s law. For example, in a number comparison task (larger of two values should be chosen), number pairs with a ratio closer to 1 are harder to process (i.e., the responses are slower and more error-prone, an effect termed ratio effect; Dehaene, 2007). Two other comparison effects are believed to reflect this ratio effect as well. The comparison distance effect (CDE; worse performance when the two to-be-compared values are numerically closer—while the sizes of the pairs are the same) and the comparison size effect (worse performance when the values are larger—while the distances of the pairs are the same) are thought to be two different ways to measure the ratio effect (Dehaene, 2007; Moyer & Landauer, 1967). In other words, these two effects can also be considered as the artifacts of the ratio effect.

**Fig. 1:**
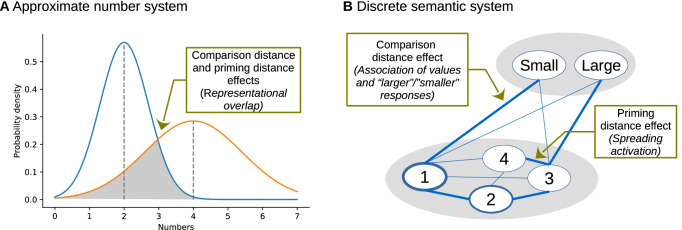
**a** A possible implementation of the approximate number system (ANS) representation. **b** The discrete semantic system (DSS) representation can explain comparison distance and priming distance effects. Note that the connectionist model of Verguts provides a functionally similar solution (Verguts et al., 2005)

An often-cited possible implementation of the ANS is a representation where the stored values are noisy, and the noise is proportional to the to-be-stored value (i.e., larger numbers are noisier; Fig. 1). In this model, the difficulty of a comparison task is proportional to the overlap between two number representations: The smaller the overlap is, the more discriminable the two values are and the easier the comparison task is. Additionally, there could be individual differences in how precise the system is (Halberda et al., 2012). Mathematically, the standard deviation of the noisy representation for a specific value (described as the Weber fraction parameter) is the precision or sensitivity of the ANS. In the model, higher sensitivity (i.e., smaller Weber fraction) leads to smaller representational overlap between the representation of two values, and e.g., to more efficient comparison performance.

This simple representation is believed to account for a series of other phenomena. Among others, the ANS model can explain the numerical priming distance effect (PDE). In the numerical PDE, the processing of a former value can enhance the processing of a later value, and the closer the two values are the stronger the priming effect is (Koechlin et al., 1999; Reynvoet & Brysbaert, 1999). According to the ANS account, the strength of the priming effect depends on the overlap of the prime and target values (Dehaene, 2004; Koechlin et al., 1999). In line with the ratio-based nature of the representation, the PDE is also an aspect of the ratio effect.

**Fig. 2:**
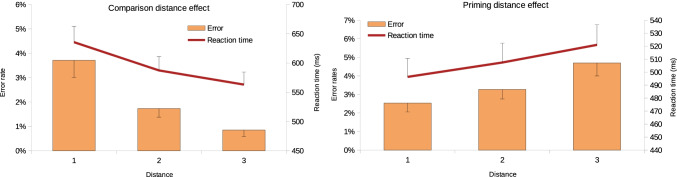
Comparison distance effect (left) and priming distance effect (right) for the error rates and reaction time. Error bars represent 95% confidence intervals

Note that while, in the CDE, the performance is worse when the distance is smaller, in the PDE, the performance is better for smaller distance (e.g., see Fig. 2 in the present results). This difference in itself may hint that the two effects do not rely on the same mechanism. However, according to a possible explanation, the single ANS may account for the opposing effect directions: When numbers are presented simultaneously, the representations may interfere, which causes worse performance with larger representational overlap and when the numbers are presented consecutively, the prime representation may help the activation of the target representation (Koechlin et al., 1999). Note that this hypothesis should be confirmed: Consecutive presentation of the numbers of a pair should reverse the distance effect in other paradigms as well. Contrarily, in comparison tasks with serially presented values of the pairs, we found CDE-like distance effect both in Arabic number comparison and in nonsymbolic dot comparison (unpublished data). Overall, an ANS model should account for the opposing directions of the CDE and PDE slopes, which account may not be entirely satisfactory at the moment.

While the ANS model is a dominant model in the numerical cognition area, there are other explanations how comparison distance and priming distance effects are generated. In an alternative model, a connectionist network may account for a series of elementary number processing phenomena (Verguts et al., 2005). Importantly, it has been proposed that while the CDE is rooted in the connections between the number nodes and the “larger” responses, the PDE is caused by the spreading activation between the number nodes (Verguts et al., 2005). In another alternative explanation, similar to the connectionist network above, the discrete semantic system (DSS) model assumes that numbers and related concepts are stored in a network of nodes and relevant effects can be generated by this simple architecture (Fig. 1, right; Krajcsi et al., 2022). In line with the connectionist model, the DSS model proposes that the CDE may be rooted in the connection of the number nodes and the “small”–“large” nodes: Smaller numbers are more strongly associated with the “small” label than the larger numbers and, in a comparison task, numbers with larger distance are easier to process because their association with “large” and “small” labels is more dissimilar. This hypothesis has been confirmed empirically in studies where the association between numbers and the “larger” response was manipulated in a comparison task and the distance effect followed the association of the numbers and the “larger” response instead of the values of the numbers (Kojouharova & Krajcsi, 2018; Krajcsi & Kojouharova, 2017). On the other hand, in the DSS model, it may be reasonable to assume that the priming effect is generated by the spreading activation between the number nodes (Fig. 1). The DSS is similar to the connectionist model by Verguts et al. (2005) in many aspects and the two models can also be considered as complementary descriptions of the same system.

To summarize, while the widely discussed ANS model assumes that the CDE and the PDE are rooted in the same mechanism (representational overlap in a noisy number representation), alternative models assume that CDE and PDE are generated by different mechanisms: CDE is the result of the connections between the values and “larger” and “smaller” labels and PDE is rooted in the spreading activation between the representations of the values.

## Empirical results on the possible common sources of number comparison and number priming

Several studies investigated the possible common causes of the CDE and PDE. A first group of relevant empirical studies investigated the correlation of the CDE and the PDE. According to the ANS account, because both the CDE and the PDE rely on the same representation and the performance in both effects depends on the Weber fraction (i.e., sensitivity) of the ANS, the correlation coefficient of the two effects ideally should be 1 (see a similar approach in Krajcsi, 2017, where the expected perfect correlation between nonsymbolic numerical comparison effects has been observed). This prediction was not confirmed by empirical studies: Significant nonzero correlation was found neither in children with symbolic numbers (Reynvoet et al., 2009) nor in adults with nonsymbolic values (Sasanguie et al., 2011). Although these results may seem to confute the ANS account, an essential limitation of correlational studies should be considered. Observed correlations are attenuated by the reliability of the variables: The noisier the variables, the smaller the observed correlation can be (Spearman, 1910). This also means that the true correlation is equal to or larger than the observed correlation, and the difference between the true and measured values depends on the reliability of the variables. If the variables have low reliability, it may completely obscure the correlation. In the cited studies, if the reliability of either the comparison or the priming distance effect is low, it is possible that while the true correlation coefficient is 1, the observed correlation is as low as 0. Importantly, Sasanguie et al. (2011) reported the reliabilities of the effects, and while the reliability of the CDE was partially acceptable (0.40 correlation between the first and second halves of the comparison task), the reliability of the PDE was very low (a nonsignificant 0.21 coefficient). This confirms that when measuring the correlation between the CDE and PDE indexes, the reliability of these indexes can be low, which can entirely obscure the true correlation. To summarize, although empirical studies demonstrate that the comparison and priming distance effects do not correlate, their results cannot be conclusive because one may not know whether the observed low correlation is the result of the low true correlation or the result of the low reliability of the variables or both.

A second group of relevant studies investigates whether the priming effect shows the ratio effect. The ANS assumes an asymmetric priming effect around the target value—that is, a prime smaller than the target should evoke a smaller priming effect compared with a larger prime with the same distance because the smaller prime has smaller noise than the larger prime, which leads to smaller representational overlap with the smaller prime than with the larger prime. In a review of several empirical works, Verguts et al. (2005) found that the PDE is symmetric in symbolic stimuli, which does not confirm the ANS account. In other words, the priming effect depends only on the distance of the prime and the target values but not on the ratio of the values, which means that while there is a PDE, it is not rooted in a possible priming ratio effect, but it is a separate effect.

A third line of evidence argues that the CDE and PDE do not rely on the same mechanism because the two effects dissociate in letter processing. It was found that while, in symbolic number comparison task, both comparison and priming distance effects can be observed, in letter comparison (i.e., is the presented letter before or after a reference letter in the alphabet), only the CDE can be observed but not the PDE (Opstal et al., 2008). Importantly, this argument assumes that number and letter comparisons rely on the same or same type of representations which assumption is backed by the similar nature of the distance effect in number and letter comparison. However, it is possible that similar effects have different generators. For example, Vigliocco et al. (2002) demonstrated a semantic distance effect in a picture naming task where a psychophysical representation (i.e., a representation obeying Weber’s law) is unlikely, while nonsymbolic number CDE most likely relies on a psychophysical representation (Krajcsi et al., 2018).

A forth type of evidence consists of a dissociation in the neural background of the CDE and PDE (Zhang et al., 2016). In an fMRI study, it was demonstrated that while the CDE relies more heavily on right parietal areas, the PDE more heavily uses left parietal areas. However, critical aspects of the tasks were admittedly not aligned: While, in the comparison task, response selection was needed (participants responded whether the first or the second number presented consecutively was larger), in the priming task, no such selection was required (participants had to press a button only if second number matched the first number). For this reason, these results may not be conclusive.

Overall, while there are several works investigating whether the PDE may be backed by the ANS or whether the CDE and the PDE are related or dissociated, there are methodological issues that question the conclusions of several of those works.

The aim of the present study is to contrast the theoretical accounts of the CDE and PDE by measuring the correlation of the CDE and the PDE slopes. To consider the possible attenuation effect, the reliability of the appropriate indexes is measured here, and we intend to ensure that they are appropriate.

## Methods

In two comparison tasks, the comparison and the priming distance effects were measured, and the correlation of these effects was calculated while the reliability of the effects was considered.

### Participants

Pilot studies (see below in the Stimuli and procedure subsection) indicated that with the planned paradigms a reliability of at least 0.51 for the CDE can be reached. Note that the present paradigm includes 4 times the number of trials compared with the pilot paradigm, therefore, we expect considerably higher reliabilities in the present data. According to the pilot study, a reliability of 0.51 for the PDE can be reached. Reliability was measured with the Spearman–Brown corrected even–odd split-half reliability values. If the true correlation coefficient is 1 as predicted by the ANS account, the observed correlation coefficient with the given reliabilities should be 0.51.^1^ To reach 95% power for this observed correlation, at least 44 participants are needed.

Eighty-four university students from various majors completed all sessions for partial course credit (64 females, *M*_age_ = 21.9 years, *SD* = 4.6 years). A few additional participants who made random guesses in any of the sessions were formerly excluded. The study was approved (201710) by the ethics committee of the Psychology Institute, ELTE Eötvös Loránd University, Hungary.

### Stimuli and procedure

In the task that measured the CDE, two single-digit numbers appeared on the two sides of the screen, and the participants had to choose the larger one by pressing the appropriate response button. In the task that measured the PDE (here, we term this task the priming task, even if both tasks included comparisons as tasks), a single-digit number appeared in the middle of the screen and participants had to choose whether the number is smaller or larger than 5 by pressing the appropriate response button. In both tasks, the numbers were visible until response. After the response, a blank screen was visible for 700 ms and an auditory feedback was given during the blank screen.

In both the comparison and the priming tasks, numbers between 1 and 4, and between 6 and 9 were used. In the comparison task, all possible number pairs with different values were presented (56 possible number pairs) 40 times, resulting in 2,240 trials. In the priming task, the same 56 number pairs were used as in the comparison task, but the numbers of a pair were presented in two trials, where the first value was later considered as the prime number and the second value was the target. In the priming task, the 112 (i.e., 56 number pairs where the two values of a pair were presented in separate trials) numbers were repeated 120 times, resulting in 13,440 trials. For both tasks, the trials within a session (see below) were randomized (with the constraint that, in the priming task, the prime–target values were presented in consecutive trials).

The whole experiment was divided into five sessions, approximately 1 hour each. The first session included the comparison task, while the remaining four sessions included the priming task, where each priming task session included trials with all 56 number pairs presented 30 times.

Although this long procedure was demanding for the participants, we used this version because it could provide acceptable reliabilities for the CDE and PDE. While, in typical paradigms that are used in the literature, the reliability of the CDE is acceptable, the reliability of the PDE is rather low (Gilmore et al., 2011; Gilmore et al., 2014; Sasanguie et al., 2011). After a series of pilots, we found that it is only the number of trials that can improve reliability further. Our preparatory studies showed that for the CDE (with all number pairs repeated 10 times instead of 40 times as used here) reliability was 0.38 for the error rates and 0.51 for the reaction time data (Spearman–Brown corrected even–odd split-half reliability). We considered that multiplying the number of trials by four will lead to a satisfying reliability for the CDE slope index. For the PDE, with similar parameters as used in the present study, the reliability was 0.79 for the error rates and 0.51 for the reaction time. In former pilot studies, we could not find appropriate parameters for the paradigm that could have led to acceptable reliability in a single session measurement of PDE, where the session could not be longer than 60 minutes to avoid fatigue.

The data were collected online on the Cognition platform (www.cognition.run), the script was written in jsPsych (de Leeuw, 2015) using the jspsych-psychophysics plugin (Kuroki, 2021). Participants received detailed instructions on how to provide optimal circumstances for the data collection. The time interval between two consecutive sessions was between 2 hours and 3 days.

### Analysis

While the distance (or the ratio) effect slope is widely used in the literature as an approximation for the ANS sensitivity, its use is not recommended in correlational studies. A main problem is that the relationship of the distance effect slope and the Weber fraction is not only nonlinear but it is nonmonotonic: Depending on the specific ratios of the stimuli and the Weber fractions of the participants, in some cases, larger distance effect slope means larger Weber fraction, while in some other cases, smaller Weber fraction (Chesney, 2018). This bias can fundamentally reduce the observed correlations (Krajcsi, 2020). (For a detailed explanation of this non-trivial relation of the effect slopes and the Weber fraction, see Chesney, 2018, and Krajcsi, 2020.) One way to overcome this issue in the present study was to use the same value-pairs in both tasks. This way since the stimuli (and the relevant ratios or distances) are the same across the two tasks and the Weber fractions of the participants are also the same in the two tasks, the Weber fractions are transformed in the same way to the CDE and PDE. Therefore, if there is a correlation between the Weber fractions behind the CDE and PDE (i.e., because they are the same Weber fraction of a single ANS), then the CDE and the PDE should correlate as well.

In the priming task, only the congruent trials (i.e., either both the prime and target numbers are smaller than 5 or both of them are larger than 5) were used, and incongruent trials were excluded from the analysis because incongruent trials show an interference effect that masks the priming effect (Reynvoet et al., 2002). The same restrictions were applied to the comparison task to avoid the issue rooted in the nonmonotonic relation between the distance effects and the Weber fraction (Chesney, 2018; Krajcsi, 2020). In the priming task, only the target trials were analyzed.

The CDE and the PDE were calculated for both the error rates and the reaction time data. Mean error rate and median reaction time for the correct responses were calculated for each distance and each participant. Distance effect slopes were calculated with linear regressions where the regressor was the distance and the outcome variable was the performance (error rate and reaction time for the CDE and PDE) for each participant. The output of these analyses was the CDE and PDE slopes for error rates and reaction times for each participant (i.e., four slope values per participant).

The reliability of the four indexes (i.e., CDE and PDE for error rates and reaction times) was investigated. It is important to highlight that even if the reliabilities of similar tasks have been reported in other works (e.g., Sasanguie et al., 2011), those results may not be relevant here because reliability depends on the specific parameters of a paradigm (e.g., the number of trials is a strong predictor) and on the population (since the commonly used test–retest correlation is a relative index of the true variance of the variable in the population’s total variance; Lindskog et al., 2013). Therefore, reliability indexes should be reported in correlational studies unless the paradigm and the population are similar to other paradigms and populations for which the reliability is known. To calculate the reliability in the present study, even-odd split-half reliability together with the Spearman–Brown prediction formula were applied (Spearman, 1910). Because the stimuli were randomized, when trials are split into even and odd trials, some conditions (in the present analysis, distances) may include more trials in the even half compared with the odd half or the other way around. Decreased number of trials in the even or the odd half of the condition can lead to lowered observed reliability. To overcome this problem, trials of a task were first sorted according to the relevant conditions (i.e., distances), and an even–odd split was applied on the ordered data, therefore, the size of the even and odd halves were equal (or the difference was only 1 if the total number of trials were odd) in all cells. Note that the split-half reliability of a multisession data is conceptually a mixture of a single-session split-half index and a test–retest reliability index: Similar to the test–retest index, it includes the variability of the changes between sessions and similar to the single-session split-half method, it splits the data not following the sessions but in a more gradual way.

Correlation for the reliability and for investigating the CDE–PDE relationship was calculated not only with the Pearson correlation coefficient, but also with Spearman’s rank correlation coefficient because the latter is not sensitive to outliers. Therefore, similar results with Pearson’s *r* and Spearman’s *r*_*s*_ denote that the results with Pearson’s *r* are not the result of some of the statistical artifacts.

For the analysis custom Python scripts, LibreOffice (Version 7.2; The Document Foundation, 2021) and CogStat (Version 2.1; Krajcsi, 2021) were used.

## Results and discussion

The raw data of the experiment reported here is available online (https://osf.io/bs94q/). The experiment was not preregistered.

CDE was observed in the comparison task, both for the error rates and reaction time (Fig. 2; for the error rate, the slope mean was −1.4% with a standard deviation of 1.3; for the reaction time, the mean slope was −36 ms with a standard deviation of 19; the slopes significantly differed from zero both in error rates, Wilcoxon signed-rank test: *T* = 23.5, *p* < .001, and in reaction times, Wilcoxon signed-rank test: *T* = 0.00, *p* < .001). Similarly, PDE was present in the priming task, both for error rates and reaction times (Fig. 2; for the error rate, the slope was 1.1% with a standard deviation of 0.9; for the reaction time, the slope was 12 ms with a standard deviation of 8; Wilcoxon signed-rank test: *T* = 94.00, *p* < .001, Wilcoxon signed-rank test: *T* = 15.00, *p* < .001, respectively).

The reliability of the four slope indexes were calculated (Table 1). All of the reliabilities were satisfying, the Spearman–Brown corrected split-half reliabilities were between 0.72 and 0.87. Similar Pearson and Spearman correlation values demonstrate that the relatively high correlations are not the result of outliers. Relying on these reliabilities, one can consider the attenuation in the prediction of the ANS model. While the ANS model predicts a true correlation value of 1 between the CDE and PDE slopes, the measured correlation should be lower because of the smaller than 1 reliabilities. According to the equation in Footnote , the expected measured correlation is 0.8 for the CDE and PDE error rate slopes (i.e.,$1\cdot \sqrt{0.868\cdot 0.738}$) and 0.78 for the CDE and PDE reaction time slopes (i.e.,$1\cdot \sqrt{0.841\cdot 0.72}$). In the following analysis, it is investigated whether the observed correlation equals these predicted correlations. Statistically, it is investigated whether the confidence intervals of the measured correlations include these predicted values (Cumming, 2014).

**Table 1:** Reliability of the CDE and PDE indexes

	Reliability—Pearson correlation	Reliability—Spearman correlation
CDE error rates	*r*_*SB*_ = .868 *r* = .767, [.661, .843]	*r*_*s*_ = .685, [.551, .784]
CDE reaction times	*r*_*SB*_ = .841 *r* = .725, [.604, .813]	*r*_*s*_ = .693, [.562, .790]
PDE error rates	*r*_*SB*_ = .738 *r* = .585, [.424, .710]	*r*_*s*_ = .527, [.353, .666]
PDE reaction times	*r*_*SB*_ = .720 *r* = .563, [.396, .694]	*r*_*s*_ = .462, [.275, .616]

Cells include the Spearman–Brown prediction correlation values for the Pearson correlation and the Pearson and Spearman correlation coefficients with 95% confidence intervals. All correlations significantly differ from zero, *p* < .001.

**Fig. 3:**
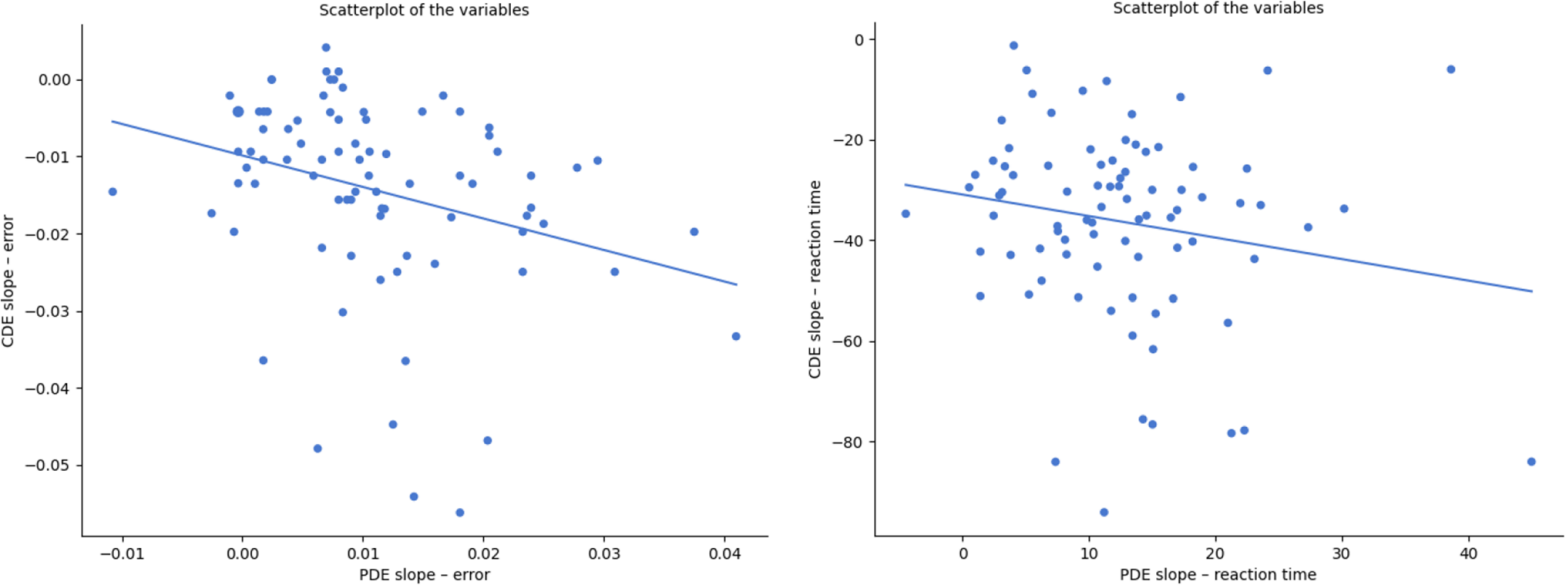
Scatter plots of the CDE and PDE in error rates (left) and reaction time (right)

The correlation values of the CDE and PDE slopes did not reach the prediction of the ANS model (see the scatter plots in Fig. 3). Although the CDE and PDE slopes for the error rates correlated significantly (i.e., the correlation coefficient differed from zero), the confidence interval did not include the predicted −0.8 value, *r*(82) = −.31, *p* = .005, [−0.488, −0.097]; *r*_*s*_(82) = −.38, *p* < .001, [−0.554, −0.186]. For the reaction-time data, the CDE and PDE slopes were not significantly different from zero, and similar to the error rates data, the confidence interval did not include the predicted −0.78 value, *r*(82) = −.19, *p* = .092, [−0.384, 0.031]; *r*_*s*_(82) = −.15, *p* = .167, [−0.355, 0.064].

The CDE was measured in a separate session, and the PDE was measured in four additional sessions. Can the fluctuation between the sessions be responsible for the observed low correlation? This possibility is not likely. The observed PDE reliability was based on a split-half index that aggregated the data of four sessions, and the relatively high PDE reliability suggests that the fluctuation between the sessions could not be high. If one assumes that the between-session fluctuation is similarly low in the CDE, then the low correlation between the CDE and PDE slopes cannot be attributed to a high fluctuation between the sessions. Alternatively, if one assumes that the fluctuation is much higher in the CDE than in the PDE, then this assumption contradicts the ANS model: The ANS account suggests that both the CDE and PDE effects rely only on the ANS sensitivity; therefore, their fluctuations cannot be different. To sum up, the results cannot be attributed to the fluctuation of the slopes between the sessions unless the ANS account is already incorrect.

While the present study carefully ensured that the reliabilities of the measured slope indexes are appropriate to observe a possible high correlation, the correlations were considerably lower than predicted by the ANS model. The present results are not in line with the ANS account of a common mechanism underlying symbolic comparison and priming.

## General discussion

The main question of the present study was whether the CDE and PDE rely on the same mechanism as predicted by the ANS model, or whether they are independent as predicted by the connectionist model of Verguts et al. (2005) or by the DSS model (Krajcsi et al., 2022). Unlike previous similar correlational studies, the current work considered the reliability of the CDE and PDE. This is critical because low reliabilities may attenuate the observed correlation, and an observed correlation around zero cannot be conclusive. Here, the reliabilities were satisfying; still, the observed correlations were considerably lower than predicted by the ANS model. These results question the ANS account of a common mechanism behind the comparison distance effect and priming distance effect. On the other hand, the results are in line with models that assume distinct mechanisms behind number comparison and number priming, such as the connectionist model of Verguts et al. (2005) or the DSS model.

In the theoretical motivation of this work, we considered the most widely cited, coherent, and mathematically relatively detailed version (Dehaene, 2007) of the ANS model. However, there are several approaches in the literature that question various details of the ANS model and provide related modifications (e.g., see the reviews of Clarke & Beck, 2021; Kadosh & Walsh, 2009; Leibovich et al., 2017). Importantly, we are not aware of any specific ANS variant that may have alternative predictions about the correlation of the CDE and PDE.

The present results are in line with former findings. Our results are in line with the symmetric priming effect (Verguts et al., 2005; i.e., that the size of the priming effect depends on the distance of the prime and the target values but not on the ratio of them). Both findings question the ANS model and confirm the network-based models of elementary symbolic number processing. The present results may be in line with additional findings, such as the dissociation of CDE and PDE in letter processing (Opstal et al., 2008), or the neural dissociation of the CDE and PDE (Zhang et al., 2016), although these latter findings may need additional confirmation. Also, a systematic empirical test is needed for any explanation that tries to account for the opposing direction of the CDE and PDE slopes, such as whether simultaneous and consecutive presentation of the numbers of a pair can change the direction of the distance effect—otherwise the opposing direction of these effects strengthen the idea that the CDE and PDE have two different generators.

Finally, it is important to highlight the essential role of reliability in correlational studies in cognitive psychology. In many cognitive studies, reliability may be low (Hedge et al., 2018). These low reliabilities attenuate the observed correlation; thus, an observed low correlation in itself cannot be conclusive: Low correlation may mean either low true correlation or low reliability or both. Moreover, when the tasks are not standardized (which is typical in cognitive psychology, where the specific stimuli or number of trials may vary between studies), the reliability may differ from what has been published in former works since reliability depends on the properties of the design. Therefore, it is essential that correlational studies should consider reliability, and if the relevant parameters of the task or the sample are unique, then reliabilities should also be reported.

## Footnotes

1 By rearranging the formula for attenuated correlation coefficients proposed by Spearman (1910), we can calculate the expected measured correlation coefficient, given the supposed true correlation and the reliabilities of the variables as ${r}_{observed}={r}_{real}\cdot \sqrt{reliability_x\cdot {reliability}_y}$, where *r*_*observed*_ is the observed correlation, *r*_*real*_ is the supposed real correlation, and *reliability*_*x*_ and *reliability*_*y*_ are the Spearman-Brown corrected split-half reliabilities of variable *X* and *Y*, respectively. Here, the expected observed correlation is $1\cdot \sqrt{0.51\cdot 0.51}$, which is 0.51.
